# Palaeolithic diet decreases fasting plasma leptin concentrations more than a diabetes diet in patients with type 2 diabetes: a randomised cross-over trial

**DOI:** 10.1186/s12933-016-0398-1

**Published:** 2016-05-23

**Authors:** Maelán Fontes-Villalba, Staffan Lindeberg, Yvonne Granfeldt, Filip K. Knop, Ashfaque A. Memon, Pedro Carrera-Bastos, Óscar Picazo, Madhvi Chanrai, Jan Sunquist, Kristina Sundquist, Tommy Jönsson

**Affiliations:** Clinical Research Centre, Faculty of Medicine, Center for Primary Health Care Research, Lund University, Malmö, Sweden; Department of Food Technology, Engineering and Nutrition, Lund University, Lund, Sweden; Center for Diabetes Research, Gentofte Hospital, University of Copenhagen, Hellerup, Denmark; NutriScience-Education and Consulting, Lda, Lisbon, Portugal; Independent researcher, London, UK; Calle José Betancort, 15, 35530 Teguise-Lanzarote, Spain

**Keywords:** Palaeolithic diet, Type 2 diabetes, Glucagon, Leptin, Lipotoxicity, Adiposopathy, Evolution

## Abstract

**Background:**

We have previously shown that a Palaeolithic diet consisting of the typical food groups that our ancestors ate during the Palaeolithic era, improves cardiovascular disease risk factors and glucose control compared to the currently recommended diabetes diet in patients with type 2 diabetes. To elucidate the mechanisms behind these effects, we evaluated fasting plasma concentrations of glucagon, insulin, incretins, ghrelin, C-peptide and adipokines from the same study.

**Methods:**

In a randomised, open-label, cross-over study, 13 patients with type 2 diabetes were randomly assigned to eat a Palaeolithic diet based on lean meat, fish, fruits, vegetables, root vegetables, eggs and nuts, or a diabetes diet designed in accordance with current diabetes dietary guidelines during two consecutive 3-month periods. The patients were recruited from primary health-care units and included three women and 10 men [age (mean ± SD) 64 ± 6 years; BMI 30 ± 7 kg/m^2^; diabetes duration 8 ± 5 years; glycated haemoglobin 6.6 ± 0.6 % (57.3 ± 6 mmol/mol)] with unaltered diabetes treatment and stable body weight for 3 months prior to the start of the study. Outcome variables included fasting plasma concentrations of leptin, adiponectin, adipsin, visfatin, resistin, glucagon, insulin, C-peptide, glucose-dependent insulinotropic polypeptide, glucagon-like peptide-1 and ghrelin. Dietary intake was evaluated by use of 4-day weighed food records.

**Results:**

Seven participants started with the Palaeolithic diet and six with the diabetes diet. The Palaeolithic diet resulted in a large effect size (Cohen’s *d* = −1.26) at lowering fasting plasma leptin levels compared to the diabetes diet [mean difference (95 % CI), −2.3 (−5.1 to 0.4) ng/ml, *p* = 0.023]. No statistically significant differences between the diets for the other variables, analysed in this study, were observed.

**Conclusions:**

Over a 3-month study period, a Palaeolithic diet resulted in reduced fasting plasma leptin levels, but did not change fasting levels of insulin, C-peptide, glucagon, incretins, ghrelin and adipokines compared to the currently recommended diabetes diet.

*Trial registration:* ClinicalTrials.gov NCT00435240

**Electronic supplementary material:**

The online version of this article (doi:10.1186/s12933-016-0398-1) contains supplementary material, which is available to authorized users.

## Background

The metabolic syndrome represents a cluster of symptoms including abdominal obesity, insulin resistance, dyslipidemia, and high fasting glucose and blood pressure [1]. The condition is associated with a fivefold increased risk of type 2 diabetes, which is characterized by insulin resistance [2] and β-cell failure [3]. Lifestyle plays a prominent role in the pathophysiology of the metabolic syndrome and type 2 diabetes [4, 5]. Specifically, an unhealthy diet with chronic caloric surplus induces hyperinsulinemia leading to ectopic lipid deposition (lipotoxicity) in the heart, liver, pancreas and muscle [6–8], increasing the risk of the metabolic syndrome, fatty liver, fatty heart and type 2 diabetes [7].

Interestingly, insulin resistance has been suggested to be a consequence and a protective mechanism against lipotoxicity [6–10]. Leptin resistance is a possible player in the roadmap to the metabolic syndrome and type 2 diabetes [7], and it has been suggested that leptin can protect against lipotoxicity [7]. Lipotoxicity can generate α-cell insulin resistance resulting in hyperglucagonaemia and increased hepatic glucose production [10]. It is increasingly recognized that the recent (in an evolutionary perspective) introduction of staple food groups such as cereal grains, dairy products and refined sugars in the human diet has occurred too recently for the human genome to have completely adapted [11].

In a previous publication from this trial, we reported significant improvements in glycated haemoglobin (HbA1c), blood lipids, blood pressure, weight and waist circumference [12] along with increased satiety [13] in patients with type 2 diabetes consuming a Palaeolithic diet, as compared to the officially recommended diet for patients with type 2 diabetes (diabetes diet) [14].

The abovementioned pathophysiological changes produce adaptations in other hormones and endocrine axes. Therefore, our aim was to investigate if the beneficial effects from a Palaeolithic diet could be tentatively explained by associated changes in adipokines, glucagon, incretins and ghrelin, and here we present new data on the fasting levels of these hormones from our previous study [12] (Additional file 1).

## Methods

Approval of the study was obtained from the regional Medical Ethics Committee and the trial was registered at ClinicalTrials.gov (Identifier: NCT00435240).

### Participants

Patients with type 2 diabetes without insulin treatment were recruited from primary health-care units in the Lund area of Sweden. Details about inclusion and exclusion criteria and patient characteristics (Table 1) have previously been published [12]. All recruited subjects were given oral and written study information prior to signing a consent form to participate in the study and were then further assessed for eligibility.

**Table 1:** Baseline characteristics

	All	Diabetes first (6/13)	Palaeolithic first (7/13)
Sex male/female (n)	10/3	4/2	6/1
Age (years)	64 (6)	63 (6)	66 (6)
Height (cm)	171 (5)	170 (6)	172 (4)
Weight (kg)	87 (17)	92 (20)	82 (13)
BMI (kg/m^2^)	30 (7)	32 (8)	28 (4)
Waist circumference (cm)	103 (14)	109 (17)	97 (9)
Diabetes duration (years)	8 (5)	11 (6)	6 (4)
Diabetic values at OGTT yes/no (n)	12/1	6/0	6/1
Lipid lowering drug (=statin) yes/no (n)	8/5	4/2	4/3
Drugs per day	4.3 (2.3)	3.7 (1.8)	4.9 (2.7)
Antihypertensive drugs per day	1.5 (1.5)	1.2 (1.2)	1.9 (1.7)
Beta-blocker yes/no (n)	4/9	1/5	3/4
Thiazide yes/no (n)	4/9	1/5	3/4
ACE inhibitor yes/no (n)	5/8	2/4	3/4
Angiotensin-II receptor blocker yes/no (n)	4/9	2/4	2/5
Calcium channel blocker yes/no (n)	3/10	1/5	2/5
Anti-diabetic drugs per day	1.2 (0.9)	1.5 (0.8)	0.9 (0.9)
Metformin yes/no (n)	9/4	5/1	4/3
Dosage, mg/day	1031 (864)	1283 (950)	814 (790)
Sulphonylurea yes/no (n)	3/10	2/4	1/6
Thiazolidinedione yes/no (n)	3/10	2/4	1/6
Plasma adiponectin (µg/ml)	4.8 (4.16)	4.8 (2.5)	4.9 (5.4)
Plasma adipsin (ng/ml)	797 (157)	804 (218)	792 (178)
Plasma C-peptide (pg/ml)	487 (275)	437 (276)	531 (289)
Plasma ghrelin (pg/ml)	568 (129)	613 (165)	530 (82)
Plasma GIP (pg/ml)	232 (91.6)	226 (83)	237 (105)
Plasma GLP-1 (pg/ml)	26.8 (3.39)	26.4 (4.5)	27.1 (2.4)
Plasma glucagon (pg/ml)	425 (44.34)	435 (52.4)	417 (38.4)
Plasma leptin (ng/ml)	9.84 (12.18)	12.1 (17)	7.9 (6.8)
Plasma resistin (ng/ml)	2.21 (0.39)	2.3 (0.4)	2.1 (0.4)
Plasma visfatin (ng/ml)	2.52 (0.75)	2.7 (0.5)	2.4 (0.7)

Data is presented as mean values with SD in brackets, unless stated

*ACE* angiotensin converting enzyme, *GIP* glucose-dependent insulinotropic polypeptide, *GLP-1* glucagon-like peptide-1

### Design

The study design and generation of random allocation sequence have been reported in detail previously [12]. In short, the study was a randomised, cross-over, dietary intervention study in which all eligible subjects were informed of the intention to compare two healthy diets and that it was not known whether one diet might be superior to the other. After randomisation, there was no blinding of dietary assignment to study participants, nor to those administering the interventions or assessing the outcomes. At study start, all subjects were randomised, by use of opaque envelopes, to start with either a diabetes diet designed in accordance with official recommendations [14] or a Palaeolithic diet [12]. Immediately after randomisation, all subjects received oral and written information about their respective initial diet individually from diabetes nurses. After 3 months, all subjects switched diets and received new oral and written information about the new diet. Advice about regular physical activity was given to all subjects.

### Diets

The information on the diabetes diet stated that it aimed to provide evenly distributed meals with an increased intake of vegetables, root vegetables, dietary fibre, whole-grain bread and other whole-grain cereal products, fruits and berries, and a decreased intake of total fat with more unsaturated fat. It was recommended that salt intake should be kept below 6 g per day. The information on the Palaeolithic diet stated that it should be based on lean meat, fish, fruit, leafy and cruciferous vegetables, root vegetables, eggs and nuts, while avoiding—as far as possible—dairy products, cereal grains, beans, refined fats, sugar, candy, soft drinks, beer and added salt. The following items were recommended in limited amounts for the Palaeolithic diet: eggs (≤2 per day), nuts (preferentially walnuts), dried fruit, potatoes (≤1 medium-sized per day), rapeseed or olive oil (≤1 tablespoon per day) and wine (≤1 glass per day). The recommended intake of the other foods was not restricted and no advice was given with regard to the proportions of food categories (e.g. animal vs. plant foods). The evolutionary rationale for a Palaeolithic diet and its potential benefits have been outlined previously [15] and detailed nutritional compositions of the diets can be found in our previous report [12].

### Procedures

An oral glucose tolerance test (OGTT) was performed in the morning after obtaining venous blood samples and measurements of blood pressure, weight and waist circumference using standard methods [12] at the start of the study, after 3 months (when switching to a new diet) and at the end of the study (after 6 months). Samples were collected in EDTA-containing tubes and centrifuged for 10 min at 4 °C. Plasma was then aliquoted and stored at −80 °C until analysis. Outcome variables in the present study included fasting plasma concentrations of leptin, adiponectin, adipsin, visfatin, resistin, glucagon, insulin, C-peptide, GIP, GLP-1 and ghrelin.

### Assessment of conditions of frozen blood samples

To assess the condition of the frozen blood samples we compared new analyses of insulin to older ones. The newly analyzed insulin values were on average 27 % lower than older analyses and the standard deviation had increased by 66 %. However, the Pearson correlation of 0.72 (adjusted R^2^ = 0.51) between new and old insulin values for the same individual and time were highly correlated (*p* < 0.0001).

### Analyses

The Bio-Plex pro™ human diabetes panel (Bio-Rad Inc., Hercules, CA, USA) a Luminex-based magnetic bead assay, was used to quantify insulin, C-peptide, ghrelin, GIP, GLP-1, glucagon, leptin, resistin and visfatin and a separate Bio-Plex assay was used to quantify adiponectin and adipsin (due to different dilution factor) in plasma according to the manufacturer’s instructions. Each run included controls of known concentration for each cytokine and a blank.

### Statistics

The statistical power calculations were based on initial primary outcomes of this intervention and previously published [12]. Data were analysed for normality (determined by Q–Q plots and the Shapiro–Wilk test in SPSS) and logarithmically transformed when necessary. If data did not show reasonable normal distribution after logarithmic transformation, the Wilcoxon matched pairs signed rank sum test was used, otherwise a paired t test was used. To analyse the difference between diets in their effects on outcomes we compared the absolute values at the end of each diet. In order to check for carry-over effects, t tests were used to compare mean values of outcome variables for the group starting with the Palaeolithic diet with those for the group starting with the diabetes diet. In order to check for period effects, t tests were used to compare the effects of the first and second diets. We performed post hoc analysis using bivariate correlations between the outcome variables presented in Table 2 and outcome variables related to glucose homeostasis and satiation. Bivariate correlations were also performed between the outcome variables presented in Table 2 and dietary variables. Outcome variables with significant correlations were entered in Simple Linear Regression. Significance was set at *p* < 0.05. All t tests were two-sided. Due to multiple outcome measures problem in this post hoc analysis a multiple outcome measures correction was made using QuickCalcs online provided by GraphPad Software (http://www.graphpad.com/quickcalcs/interpretPValue1/). Statistical analysis was performed with SPSS for Mac Version 20 (IBM SPSS Statistics for Mac, Version 20.0, IBM Corp., Armonk, NY, USA).

**Table 2:** Hormone levels, and weight, after the Palaeolithic diet and diabetes diet

Outcome	Palaeolithic diet	Diabetes diet	Delta diets	*p* ^a^
Adiponectin (µg/ml)	5.2 ± 4.4 (2.5 to 7.9)	5.7 ± 5.4 (2.5 to 9.1)	−0.5 ± 1.2 (−1.3 to 0.2)	0.153
Adipsin (ng/ml)	787 ± 182 (677 to 896)	776 ± 153 (684 to 869)	10 ± 79 (−37 to 58)	0.650
C-peptide (pg/ml)	455 ± 224 (319 to 590)	412 ± 204 (289 to 535)	43 ± 262 (−116 to 201)	0.644
Ghrelin (pg/ml)	540 ± 97 (481 to 598)	566 ± 145 (478 to 654)	−26 ± 74 (−70 to 18)	0.226
GIP (pg/ml)	254 ± 266 (93 to 415)	186 ± 75 (141 to 232)	68 ± 264 (−92 to 227)	0.600^b^
GLP-1 (pg/ml)	27 ± 9.3 (22 to 33)	27 ± 3.7 (25 to 29)	0.4 ± 7.7 (−4.3 to 5.12)	0.235^b^
Glucagon (pg/ml)	409 ± 40 (385 to 433)	431 ± 51 (400 to 463)	−22 ± 43 (−48 to 3.9)	0.089
Insulin (pg/ml)	248 ± 138 (165 to 332)	336 ± 327 (138 to 533)	−87 ± 240 (−232 to 58)	0.266
Insulin^c^ (pg/ml)	401 ± 174 (296 to 506)	391 ± 115 (322 to 461)	9.8 ± 172 (−94 to 114)	0.840
Leptin (ng/ml)	5.1 ± 4.9 (2.1 to 8.0)	7.4 ± 8.3 (2.4 to 12)	−2.3 ± 4.6 (−5.1 to 0.4)	*0.023* ^b^
Resistin (ng/ml)	2.5 ± 0.9 (1.9 to 3.0)	2.3 ± 0.6 (2.0 to 2.7)	0.2 ± 0.6 (−0.2 to 0.5)	0.356
Visfatin (ng/ml)	2.4 ± 0.7 (2.0 to 2.9)	2.5 ± 0.6 (2.1 to 2.8)	−0.1 ± 0.5 (−0.3 to 0.3)	0.906
Weight (kg)	81 ± 13 (74 to 88)	84 ± 15 (76 to 92)	−3.3 ± 3.8 (−5.7 to −1.0)	*0.008*

Data are mean ± standard deviation (95 % CI)

Significance tests are paired t test for normally distributed data and Wilcoxon matched pairs signed rank sum test for non-normally distributed data

Significant *p* values are indicated by italics font

*GIP* glucose-dependent insulinotropic polypeptide, *GLP-1* glucagon-like peptide-1

^a^ *p* for difference between diets

^b^Data non-normally distributed (Wilcoxon matched pairs signed rank sum test)

^c^Old insulin values previously published

## Results

### Participant flow

**Fig. 1:**
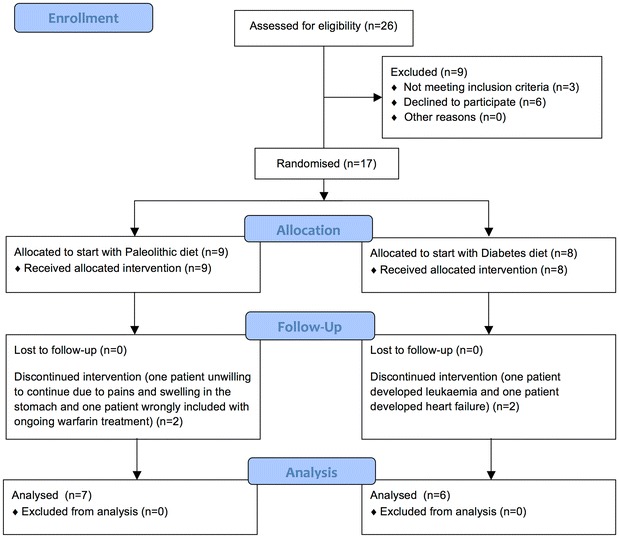
Flow diagram of participant recruitment

All reported analyses are per protocol analyses on the 13 participants (3 women, 10 men) who completed the trial (Fig. 1). Four subjects were excluded for the following reasons: one starting with Paleolithic diet was wrongly included with ongoing warfarin treatment, one starting with Paleolithic diet was unwilling to continue due to abdominal pains and bloating, one starting with diabetes diet was excluded after developing leukemia, and one starting with diabetes diet was excluded after developing heart failure. Dates defining the periods of recruitment and follow-up, and side effects have been previously published [12].

### Baseline data

Individual characteristics regarding anthropometric measurements, medication and outcome variables have been reported in detail previously [12] and are summarised in Table 1. The participants starting with the Palaeolithic diet compared to those starting with the diabetes diet did not differ at baseline for any of the outcome variables (Table 1). No carry-over or period effect was observed.

### Outcomes

**Fig. 2:**
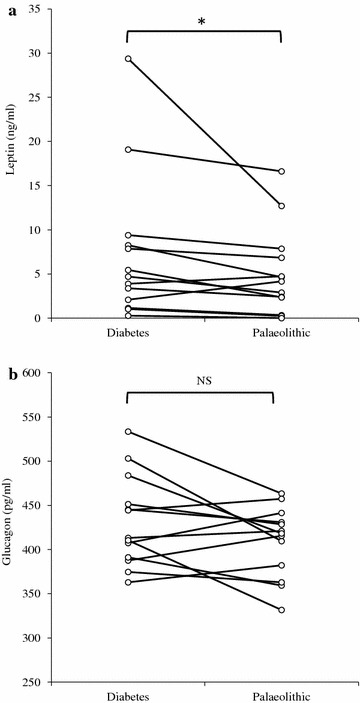
Fasting hormone levels after the Palaeolithic diet and diabetes diet for leptin and glucagon. Data show individual differences in **a** leptin and **b** glucagon after 3 months in response to the Palaeolithic and diabetes diets. Significance of the difference is indicated by *asterisks* (*p* < 0.05). *NS* non-significant

The absolute level of plasma leptin after the Palaeolithic diet was lower than after the diabetes diet (large effect size, Cohen’s *d* = −1.26; *p* = 0.023) (Table 2; Fig. 2) [16, 17]. When one outlier (more than 3 SDs) was excluded, the mean difference of leptin after the diets was normally distributed and the difference remained significant (*p* = 0.031). However, due to multiple outcome measures problem the probabilities of having a *p* value less than 0.023 just by chance in our dataset is 20.8 %.

The absolute level of glucagon at the end of the Palaeolithic diet was lower than at the end of the diabetes diet (moderate effect size, Cohen’s *d* = −0.51), but this difference did not reach statistical significance (*p* = 0.089) (Table 2; Fig. 2).

As previously reported, weight loss was significantly greater (−3.3 kg) after the Palaeolithic diet than the diabetes diet (*p* = 0.008).

No statistically significant differences between the diets for the other variables were observed (Table 2).

#### Correlations and linear regression

In post hoc analysis of within-subject differences (value after the Palaeolithic diet minus value after the diabetes diet) we found that leptin correlated with fasting plasma insulin (Spearman’s correlation 0.55, *p* = 0.049), grams of dietary fat (Spearman’s correlation −0.66, *p* = 0.013), percentage of dietary fat (Spearman’s correlation −0.55, *p* = 0.049), grams of dietary saturated fat (Spearman’s correlation −0.59, *p* = 0.033), grams of dietary fatty acid C16:0 (Spearman’s correlation −0.57, *p* = 0.041), and grams of dietary fatty acid C18:0 (Spearman’s correlation −0.55, *p* = 0.049); glucagon correlated with area under the curve (AUC) for insulin_0–120 min_ (Pearson’s correlation 0.94, *p* = 0.015), stimulated AUC insulin_0–120 min_ (Pearson’s correlation 0.55, *p* = 0.047), fasting plasma insulin (Pearson’s correlation 0.63, *p* = 0.019), satiety quotient for dietary glycaemic index per meal (Pearson’s correlation −0.56, *p* = 0.045), dietary glycaemic load (Pearson’s correlation 0.63, *p* = 0.021), dietary glycaemic index (Pearson’s correlation 0.73, *p* = 0.005), dietary fatty acid C20:5 (EPA) (Pearson’s correlation 0.58, *p* = 0.037), dietary fatty acid C22:6 (DHA) (Pearson’s correlation 0.57, *p* = 0.04) and dietary vitamin B12 (Pearson’s correlation 0.57, *p* = 0.041) (Table 3).

**Table 3:** Exploratory analysis

	Pearson’s correlation r^a^	Spearman’s correlation r^b^	Adjusted R^2^	*p* ^c^
Leptin (ng/ml) versus				
Fasting insulin (ng/ml)		0.555		0.049
Fat (g)		−0.665		0.013
Fat (%)		−0.555		0.049
SAF (g)		−0.593		0.033
Fatty acid C16:0 (g)		−0.571		0.041
Fatty acid C18:0 (g)		−0.555		0.049
Glucagon (pg/ml) versus				
AUC insulin_0-120_ (nmol/l min)	0.946		0.383	0.015
Stimulated AUC insulin_0-120_ (nmol/l min)	0.558		0.249	0.047
Fasting plasma insulin (pmol/l)	0.637		0.352	0.019
Satiety quotient for glycaemic index per meal (RS)	−0.562		0.254	0.045
Glycaemic load (g)	0.63		0.342	0.021
Glycaemic index	0.731		0.491	0.005
Fatty acid C20:5, n-3, EPA (g)	0.581		0.277	0.037
Fatty acid C22:6, n-3, DHA (g)	0.575		0.27	0.04
Vitamin B-12 (µg)	0.571		0.265	0.041
Adipsin (ng/ml) versus				
Satiety quotient for GL per meal (RS/kg)	0.581		0.277	0.037
GIP (pg/ml) versus				
Fasting plasma insulin (pmol/l)		0.555		0.049
GLP-1 (pg/ml) versus				
Stimulated AUC insulin (nmol/l min)		0.654		0.015
AUC insulin (nmol/l min)		0.67		0.012
Resistin (ng/ml) versus				
Fasting plasma insulin (pmol/l)	−0.728		0.451	0.041
Satiety quotient for GL per meal (RS/kg)	0.810		0.598	0.015
Visfatin (ng/ml) versus				
Fasting plasma glucose (mmol/l)	−0.557		0.248	0.048

Exploratory analysis was conducted to check for significant correlations between the outcome variables presented in Table 2 and outcome variables related to glucose homeostasis and satiation. Bivariate correlations were also performed between the outcome variables presented in Table 2 and dietary variables. This analysis consisted in bivariate Pearson or Spearman’s (for non normally distributed variables) correlation between within-subject differences in outcome and dietary variables. Normally distributed outcomes that were significant in Pearson’s correlation were entered into simple linear regression

^a^Pearson’s correlation for normally distributed variables

^b^Spearman’s correlation for non normally distributed variables

^c^ *p* value for bivariate correlation and simple linear regression

## Discussion

This small trial showed that a Palaeolithic diet decreased fasting plasma leptin, but did not affect fasting levels of insulin, C-peptide, glucagon, incretins, ghrelin and adipokines significantly compared to the currently recommended diabetes diet.

Weight loss interventions have been shown to decrease leptin concentrations [18], and in our trial leptin decreased only with the intervention that induced weight loss, i.e. the Palaeolithic diet. However, post hoc analysis revealed no correlation between difference in weight loss and leptin after the diets (Spearman’s correlation 0.11, *p* = 0.721).

Interestingly, genetic and in vitro studies indicate insufficient adaptation of the human leptin system to a diet based on cereal grains [19, 20]. Therefore cereal grains could hypothetically lead to leptin resistance and higher leptin values. Our finding of lower leptin following a Palaeolithic diet virtually devoid of cereal grains compared to a diabetes diet with cereal grains supports this notion, and could represent the mechanism behind our previous findings of improved glucose control and blood lipids [12] and greater satiety per calorie from the Palaeolithic diet [13].

In our study there was a non-significant lower fasting glucagon levels after the Palaeolithic diet compared to the diabetes diet, which could be a result of the amelioration of leptin sensitivity in the pancreatic islets. However, this hypothesis should be tested in trials with adequate statistical power.

Due to the small sample size, we were not able to conduct a multivariate analysis adjusting for weight loss to explore the independent effect of the Palaeolithic diet on leptin and glucagon. Therefore, our results might be explained by the weight loss produced only during the Palaeolithic diet, as already mentioned.

Insulin plays a central role in type 2 diabetes, but despite this we found no difference in fasting insulin between the diets. Compared to baseline, there was a significant decrease in insulin (*p* = 0.004 and 0.023, for old and new insulin analysis, respectively) after the Palaeolithic diet, which may be explained by weight loss.

Adiponectin appears to play an important role in type 2 diabetes due to its anti-inflammation, antiatherogenic, and insulin-sensitizing properties [21], yet we found no difference between the diets. However, there is some controversy regarding the beneficial effects of adiponectin in type 2 diabetes [22–25].

In the exploratory analysis there was a positive correlation between change in fasting leptin and insulin, which could be explained by the mechanisms discussed above and recently reviewed by Nolan et al. [8]. This finding is consistent with other studies where a positive correlation between fasting leptin and insulin has also been shown [26, 27]. It has been shown that treatment with recombinant human leptin does not improve insulin sensitivity in obese patients with type 2 diabetes [28], contrary to what happens in patients with severe leptin deficiency [29]. This might support the notion that patients with type 2 diabetes suffer from leptin resistance. Interestingly, within-subject differences in fasting leptin correlated negatively with the intake of total fat (in grams and percent) and C16:0 and C18:0 fatty acids. These results are consistent with a randomised controlled trial where a low-fat diet lowered leptin levels more than a high-fat diet [30]. On the other hand, in a well controlled study leptin levels were higher with a low-fat diet than a low-glycaemic index or very low–carbohydrate diet [31]. Other trials found no effect of fat restriction on leptin levels [32, 33]. This inconsistency in results may be due to differences between individuals in gene variants related to leptin physiology [34].

### Comparison with findings from other studies

In a previous trial from our group, leptin decreased significantly during Palaeolithic and Mediterranean diets, respectively, with no differences between diets, but after exclusion of one outlier with a high grain intake in the Palaeolithic diet group there was a significantly greater decrease in leptin in this group [35]. Interestingly, in the same study there was a strong correlation (Pearson’s correlation 0.50, *p* = 0.008) between change in leptin and intake of cereals [35]. Nevertheless, contrary to our previous finding there was no correlation in the data (Spearman’s correlation 0.22, *p* = 0471). Additionally, data from the present trial indicated that the Palaeolithic diet is more satiating than the diabetes diet [13], consistent with another trial from our group [35]. Other randomised clinical trials have shown beneficial effects of a Palaeolithic diet compared with other healthy diets on cardiovascular risk factors [36] and body fat [37]. A recent systematic review and meta-analysis, where these studies were included [12, 36–38], showed that a Palaeolithic diet improves some components of the metabolic syndrome more than the healthy control diets [39].

The Mediterranean diet has been the focus of several publications regarding its role in the metabolic syndrome and type 2 diabetes [40–43]. A systematic review and meta-analysis showed that the Mediterranean diet was superior to control diets for all components of the metabolic syndrome [44]. Another systematic review and meta-analysis concluded that the Mediterranean diet decreased HbA1c, but not fasting glucose, more than control diets but not more than the Palaeolithic diet [42]. An important consideration with respect to the characteristics of Mediterranean and Palaeolithic diets concerns their resemblance. Both emphasize a high intake of whole unprocessed foods, specifically: fruits, vegetables, fish, nuts, and olive oil, while the limitation in the intake of wholegrain cereals and legumes in the Paleolithic diet is the main difference. In light of the role that inflammation and oxidative stress might play in glycaemic control in type 2 diabetes [45], potential mechanisms behind the beneficial effects of the Mediterranean and Palaeolithic diets could be attributed to their anti-oxidative and anti-inflammatory capacity [41, 46]. Thus, both the Mediterranean diet and the Palaeolithic diet share common features that render them as healthy options in patients with type 2 diabetes, and represent a step forward for an optimal human diet.

Vegetarian diets are regarded as a healthy option for western diseases as well. A recent systematic review and meta-analysis investigated the effects of a vegetarian diet on glycemic control in type 2 diabetes [47], resulting in better HbA1c, but not fasting glucose, than control diets. None of the the included trials tested a vegetarian diet against a Palaeolithic or Mediterranean diet.

Importantly, a systematic review and meta-analysis assessed the effect of various diets on glycemic control in type 2 diabetes [48]. The results indicate that all the diets, namely low-carbohydrate, low-glycaemic index, Mediterranean and high-protein diets, improved HbA1c compared with their respective control diets. Consequently, the best dietary approach for the management of type 2 diabetes continues to be a matter of debate.

## Limitations of the present study

A limitation of this study, as with most other dietary trials, is the lack of blinding after randomisation. To minimise this problem, all study participants were informed of the intention to compare the effect of two healthy diets for the treatment of type 2 diabetes and that it was not known which one would be superior. Also, written information with dietary advice, food recipes and behavioural support were similarly formulated for both diets. The difference in weight loss, macronutrient composition and glycaemic load between the diets precludes a definite conclusion about the specific role of different food choices on the endocrine system.

The results of this study should be interpreted with caution for other reasons as well. First, we have the limitation of multiple outcome measures problem and the probability of type I error for leptin in our study is 20.8 %. Secondly, the outcomes generated from this post hoc analysis represent exploratory investigations; the primary outcomes have been previously published. Lastly, this study has a small sample size which precludes us from performing adjusted multivariate analysis. This is specially relevant for weight loss because it decreased only during the diabetes diet and the difference after the diets was 3.3 kg (*p* = 0.008). As a result, since weight loss is a principal driver of improved leptin sensitivity we are not certain about the independent effect of the diets on the results.

## Conclusion

We show that a Palaeolithic diet results in significantly lower fasting plasma leptin, non-significantly lower fasting plasma glucagon concentrations as well as weight loss, compared to a standard diabetes diet. Human beings are well adapted to food groups similar to those found in the Palaeolithic era during our evolution, and, hypothetically, the lower leptin and glucagon levels could indicate that deviations from this template is not optimal and could explain our previously reported findings on glucose control, blood lipids, blood pressure and satiety. But the small sample size of the present study makes it impossible to perform adjusted multivariate analysis and the observed weight loss after the Palaeolithic diet may also contribute to explain our results. Long-term and adequately powered trials investigating the effects of Palaeolithic diet are warranted.

## Additional file

10.1186/s12933-016-0398-1 Supporting data set. Data set supporting our results on hormones (adipokines, glucagon and incretins), post hoc analysis and previously published outcomes (worksheet ‘Data set’). Each row numbered 1-13 in first column ‘Person-ID’ corresponds to data from one participant. Each column heading contains a variable name stating what individual mean has been measured, in what unit and whether on the Paleolithic diet, diabetes diet or if it is the difference between the two diets (DeltaPd, value during Paleolithic diet minus value during diabetes diet). The variable names and their description are listed below.

## Abbreviations

AUC: area under the curve
GIP: glucose-dependent insulinotropic polypeptide
GLP-1: glucagon-like peptide-1
HbA1c: glycated haemoglobin
OGTT: oral glucose tolerance test
